# Geographic Distance and Social Isolation Among Family Caregivers Providing Care to Older Adults

**DOI:** 10.1093/geroni/igaa057.078

**Published:** 2020-12-16

**Authors:** Lun Li, Andrew Wister

**Affiliations:** Simon Fraser University, Vancouver, British Columbia, Canada

## Abstract

Family caregiving is associated with social isolation, but the role of geographic distance between caregiver and receiver in caregiving experience is unclear with mixed research findings. This study examined the relationship between geographic distance and caregiver social isolation (CSI), and explored the interaction between geographic distance and caregiving intensity in association with CSI. Based on the Ecological Model of Caregiver Isolation, hierarchical linear regression and ANCOVA analyses were applied to conduct data analysis with the 2012 Canadian General Social Survey (N=2,881). Caregivers living a short distance from receivers reported the lowest CSI than coresident, moderate and long distance caregivers. Being involved in higher intensity caregiving as the primary caregiver, undertaking more caregiving tasks, and providing care more frequently resulted in higher CSI scores. Additionally, long and moderate distance caregivers reported greater CSI than coresident and short distance caregivers only when providing higher intensity caregiving. Geographic distance is a salient contextual factor affecting CSI, and longer distance creates environmental barriers for caregiving provision. Employing a granulated measure of geographic distance positioned within an ecological framework facilitates an understanding of the nuanced association between geographic proximity and CSI. Furthermore, the identified interactive effects between geographic distance and caregiving intensity on CSI further reveal the complexity of caregiving experience. The findings are relevant for programs supporting caregivers in different contexts, especially physical distance.

